# Supplementary material for: Get the happiness out–An online experiment on the causal effects of positive emotions on giving
Source: PLoS One. 2023 Aug 23;18(8):e0290283. doi: 10.1371/journal.pone.0290283 (PMC10446232; doi:10.1371/journal.pone.0290283)
Supplement: S1 Appendix — (DOCX) [file pone.0290283.s001.docx]

ONLINE APPENDIX

Appendix A.


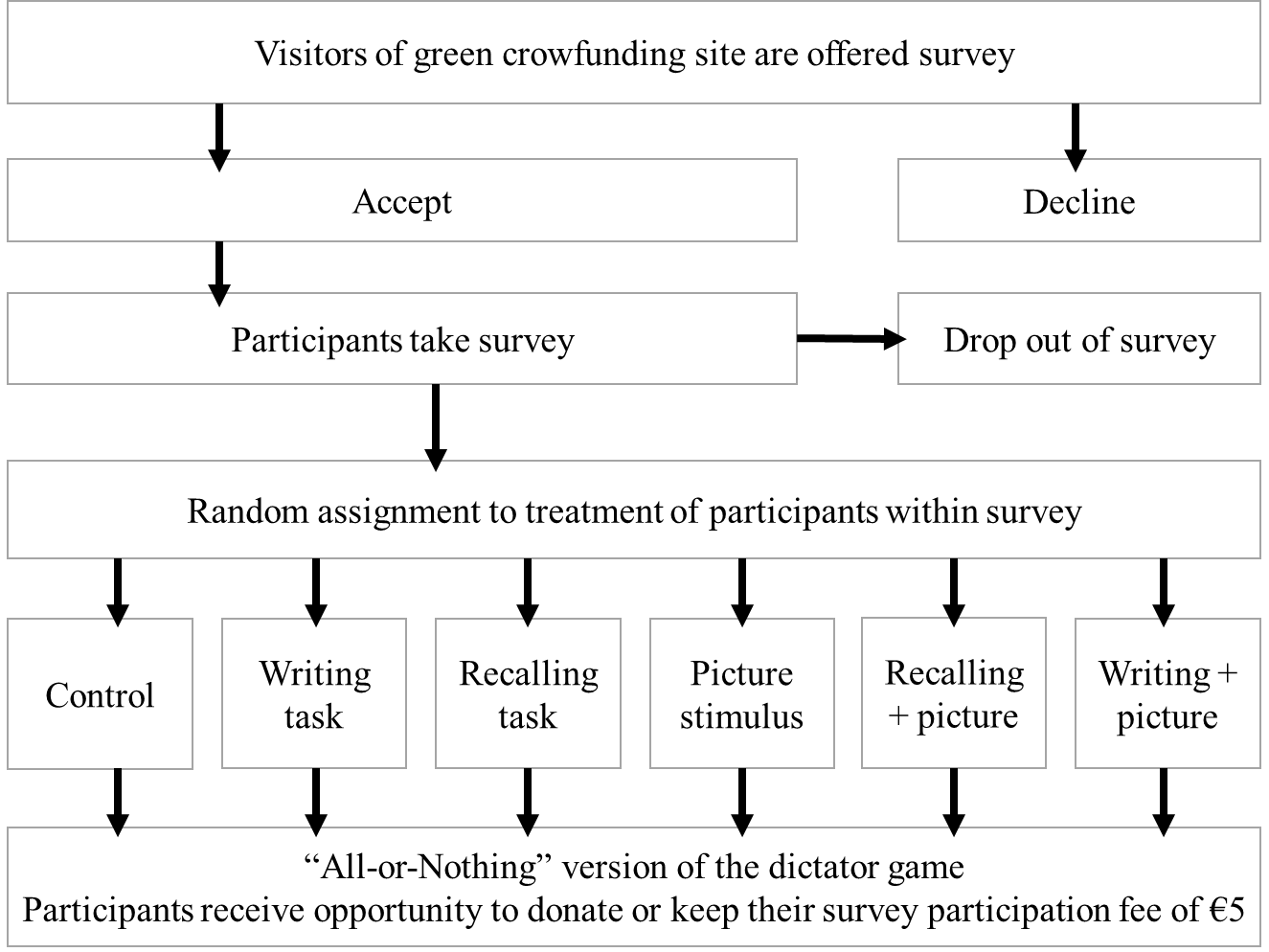
*Figure A.1: Experimental design*

Appendix B. Questionnaire (translated into English)

*[Intro]*

As XXX, we would like to know our supporters better to convince even more people of our idea of a sustainable world in the future.

For this purpose, we are working together with a team of researchers from the University of XXX. Together we have designed a short online survey in which we ask you to participate. Participation will take between 3 and 5 minutes. You will receive compensation of 5€ for participation.

We assure you that this study and the associated data serve purely scientific purposes. Of course, data protection laws will be observed, and your data will not be passed on to third parties.

Welcome to our study, and thank you for your interest. Please start by answering a few questions about yourself.

*[Page 1]*

*Question for variable ‘Age’:*

Welcome to our study, and thank you for your interest. First, we would like to ask a few questions about yourself.

Please enter your age: ______ years

*Question for variable ‘Female‘:*

Please indicate your gender:

□ Male

□ Female

*Question for variable ‘Berlin community’:*

Please indicate your zip code and the name of your primary residence.? _____ _____

*The following items are relevant to construct the variable ‘Education’:*

What is the highest school or college degree you have completed?

□ I left school without a diploma

□ I am currently going to school

□ I am currently studying

□ Volks- / Hauptschulabschluss (GDR: 8th grade)

□ Realschulabschluss / Mittlere Reife (GDR: 10th grade)

□ Fachhochschulreife (completion of a specialized secondary school)

□ Abitur/university entrance qualification

□ Fachhochschule or Berufsakademie degree (GDR: engineering and technical college degree)

□ University or college degree

□ Doctorate or habilitation

□ Other degree, namely: _______________

*[Page 2]*

Next, we would like to ask you some questions about your attitudes and individual preferences.

How would you rate your willingness to take risks in the following areas? How is it …

|  | Not at all willing to take risks |  |  |  |  |  |  |  |  |  | Fully prepared to take risks |
| --- | --- | --- | --- | --- | --- | --- | --- | --- | --- | --- | --- |
|  | 0 | 1 | 2 | 3 | 4 | 5 | 6 | 7 | 8 | 9 | 10 |
| In general | □ | □ | □ | □ | □ | □ | □ | □ | □ | □ | □ |
| Finance | □ | □ | □ | □ | □ | □ | □ | □ | □ | □ | □ |
| Faith in other people | □ | □ | □ | □ | □ | □ | □ | □ | □ | □ | □ |

*The following 6 items are relevant to construct the index variable ‘Environmental concern’:*

How strongly do you agree to the following statements?:

|  | Fully disagree | Rather disagree | Neither disagree nor agree | Rather agree | Fully agree |
| --- | --- | --- | --- | --- | --- |
| Humans have the right to modify the natural environment to suit their needs | □ | □ | □ | □ | □ |
| Humans are severely abusing the planet | □ | □ | □ | □ | □ |
| Plants and animals have the same right to exist as humans | □ | □ | □ | □ | □ |
| Nature is strong enough to cope with the impacts of modern industrial nations | □ | □ | □ | □ | □ |
| Humans were meant to rule over the rest of nature | □ | □ | □ | □ | □ |
| The balance of nature is very delicate and easily upset | □ | □ | □ | □ | □ |

*[Page 3]*

*Question for variable ‘Crowdfunding in 2017’:*

Now please answer a few questions about your engagement on crowdfunding platforms over the past year.

In 2017, have you engaged in a crowdfunding platform that offers social projects or environmental protection projects? These can be higher amounts, but also smaller ones.

□ Yes

□ No

*[Filter: If question 4 "Yes"]*

What was the total amount you donated to such projects in 2017? If you do not remember exactly, please estimate.

_____Euro

*[Page 4]*

Now please answer a few questions about your social environment.

What would you say: How many close friends do you have? ___ Number of friends

How would you describe your position in your (close) circle of friends?

□ You have a large circle of friends in which many friends know each other

□ You have individual friendships in which your friends (usually) do not know each other

□ You have several circles of friends in which your friends know each other

□ I have no friends

On the following list, you find people who may be important to you in some way.

How about you when it comes to the following?

*Dropdown list: Partner, Family, Friends / Acquaintances, Nobody,*

To whom do you share personal thoughts and feelings or talk about things you would not tell everyone?

(1)_________ (2)_________ (3)_________

*Question for variable ‘Signaling’ and ‘Signaling context’:*

In general, who do you talk to about having contributed financially to a crowdfunding project?

(1)_________ (2)_________ (3)_________

Who do you talk to in advance about making a financial contribution to a crowdfunding project?

(1)_________ (2)_________ (3)_________

*[Page 5]*

*The variables ‘Feeling responsible’, ‘Local identification’, ‘Warm glow feelings, ‘Reward seeking’, ’Expectation society’, and ‘Behavior of family and friends are based on the following questions:*

How strongly do you agree to the following statements?

|  | Fully disagree | Rather disagree | Neither disagree nor agree | Rather agree | Fully agree |
| --- | --- | --- | --- | --- | --- |
| I feel responsible for contributing to sustainable projects and startups | □ | □ | □ | □ | □ |
| I support sustainable projects and startups, as it benefits my region | □ | □ | □ | □ | □ |
| It makes me feel good to contribute to sustainable projects and startups | □ | □ | □ | □ | □ |
| I support sustainable projects and startups because I am interested in the exchange item | □ | □ | □ | □ | □ |
| Society expects me to support sustainable projects and startups | □ | □ | □ | □ | □ |
| My environment (friends, family, colleagues) support sustainable projects and startups | □ | □ | □ | □ | □ |

*[Page 6]*

*[Text control group]*

Done!

You have answered all the questions. By participating in the survey, you have earned 5€. If you like, you can donate the amount to XXX, the environmental organization XXX, or receive a personal voucher for the sustainable online store XXX.

*[Text treatment groups]*

You have answered (almost) all the questions. By taking part, you have earned 5€. If you like, you can donate the amount to XXX, the environmental organization XXX, or receive a personal voucher for the sustainable online store XXX.

*[Treatment 1: Writing task]*

Before deciding, we are interested in understanding the daily experiences that make you happy or cheerful. Please describe an event in the past year that made you happy? For example, this can be anything, the birth of a child, the marriage of a relative, or success in your job.

*[Treatment 2: Recalling task]*

Before deciding, we are interested in understanding the daily experiences that make you happy or cheerful. For example, this can be anything, the birth of a child, the marriage of a relative, or success in your job. Please think of an event in the past year that made you happy?

*[Treatment 3: Picture]*


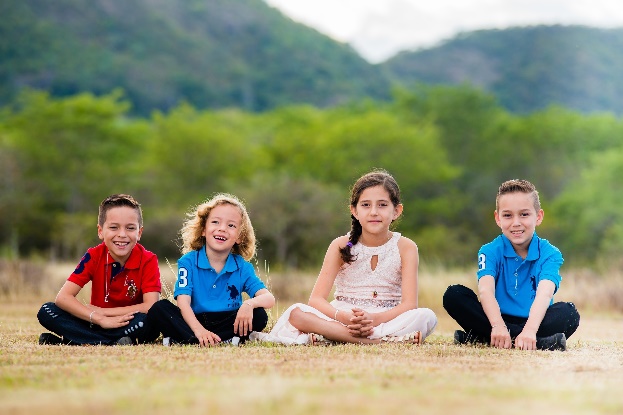


*[Treatment 4: Recalling task plus picture]*

Before deciding, we are interested in understanding the daily experiences that make you happy or cheerful. For example, this can be anything, the birth of a child, the marriage of a relative, or success in your job. Please think of an event in the past year that made you happy?

*[Treatment 5: Writing task plus picture]*

Before deciding, we are interested in understanding the daily experiences that make you happy or cheerful. Please describe an event in the past year that made you happy? For example, this can be anything, the birth of a child, the marriage of a relative, or success in your job.

*[Choice - randomly appearing]*

What do you want to do with your earned money?

- Donate to the environmental organization XXX.
- Receive a personal voucher.
- Donate to the sustainable crowdfunding platform XXX.

Thank you for taking part in the survey.

I agree with the terms of participation and the privacy policy.

*[Page 7 for those who choose the voucher]*

Please provide us with your email address so that we can forward your voucher to you.

Thank you for taking part in the survey.

I agree with the terms of participation and the privacy policy.

Appendix C. Definition of variables and summary statistics

| Variables | Description | Mean | Standard deviation |
| --- | --- | --- | --- |
| *Outcome giving experiment* |  |  |  |
| Contribution behavior | Categorical variable includes three mutually exclusive alternatives of contributing in the in the "All-or-nothing" dictator game. |  |  |
| Contribution to the crowdfunding platform | Base category, 1 if the respondent contributes to the crowdfunding platform | 0.62 | 0.49 |
| Contribution to the environmental organization | 1 if the respondent contributes to environmental organization | 0.29 | 0.45 |
| No Contribution | 1 if the respondent prefers the private voucher | 0.09 | 0.29 |
| *Treatment variables* |  |  |  |
| Positive emotions | 1 if the respondent receives a treatment. | 0.83 | 0.38 |
| Writing task | 1 if the respondent receives the writing task. |  |  |
| Recalling task | 1 if the respondent receives the recalling task |  |  |
| Picture | 1 if the respondent receives the picture. |  |  |
| Recalling task plus picture | 1 if the respondent receives the recalling task plus picture. |  |  |
| Writing task plus picture | 1 if the respondent receives the writing task plus picture. |  |  |

|  | **Definition of variables and summary statistics** *– Continued* | | |  |  | |
| --- | --- | --- | --- | --- | --- | --- |
| Variables | Description | | | Mean | Standard deviation | |
| *Observational data* |  |  |  |  |  |  |
| *Motivations related to reputational concerns* |  | | |  |  | |
| Expectation society | 1 if the subject’s response to the statement “The society expects me to support sustainable projects and startups” (1 = fully disagree to 5 = fully agree) is greater than or equal to the category “rather agree”. | | | 0.17 | 0.38 |  |
| Behavior of family and friends | 1 if the subject’s response to the statement “My environment (friends, family, colleagues) support sustainable projects and startups” (1 = fully disagree to 5 = fully agree) is greater than or equal to the category “rather agree”. | | | 0.34 | 0.48 |  |
| *Benefits of giving* |  | | |  |  | |
| Reward seeking | 1 if the subject’s response to the statement “I support sustainable projects and startups because I am interested in the exchange goods” (1 = fully disagree to 5 = fully agree) is greater than or equal to the category “rather agree | | | 0.24 | 0.43 | |
| *Psychological benefits of giving* |  | | |  |  | |
| Warm glow feelings | 1 if the subject’s response to the statement “It makes me feel good to contribute to sustainable projects and startups” (1 = fully disagree to 5 = fully agree) is greater than or equal to the category “rather agree”. | | | 0.82 | 0.38 | |
| *Values and attitudes related to giving* |  | | |  |  | |
| Feeling responsible | 1 if the subject’s response to the statement “I feel responsible for contributing to sustainable projects and startups” (1 = fully disagree to 5 = fully agree) is greater than or equal to the category “rather agree”. | | | 0.72 | 0.45 | |
| Local identification | 1 if the subject’s response to the statement “I support sustainable projects and startups as it benefits my region” (1 = fully disagree to 5 = fully agree) is greater than or equal to the category “rather agree”. | | | 0.49 | 0.50 | |

|  | **Definition of variables and summary statistics** *– Continued* | |  |  |  |
| --- | --- | --- | --- | --- | --- |
| Variables | Description | | Mean | Standard deviation |  |
| Social trust | | Domain specific risk question on social trust based on the question “How would you rate your willingness to take risks in the following areas? How is it with ... "Your faith in other people?" on an 11-point Likert scale ranging from "not at all willing to take risks" to "very willing to take risks". The question is based on the experimentally validated survey questions from the German Socio-Economic Panel (SOEP) (Dohmen et al. 2011; Dohmen et al., 2012). | 5.59 |  | |
| *Green values* | |  |  |  | |
| Environmental concern | | Additive score based on six standardized statements on the original New Ecological Paradigm (NEP) scale (Dunlap et al., 2000).  (1) “Humans have the right to modify the natural environment to suit their needs”;  (2) “Humans are severely abusing the planet”; (3) “Plants and animals have the same right to exist as humans”; (4) “Nature is strong enough to cope with the impacts of modern industrial nations”; (5) “Humans were meant to rule over the rest of nature”; and (6) “The balance of nature is very delicate and easily upset”.  The underlying question is “How strongly do you agree with the following statement?” and the five ordered response categories are “fully disagree”, “rather agree”, “neither disagree nor disagree”, “rather agree”, and “fully disagree”. The variable is designed by constructing dummy variables that equal 1 if the respondent agrees with the respective statement rather or fully (in the case of positively keying items) or rather or fully disagree weakly (in the case of negatively keying items), respectively. The six dummy variables are added up. | 4.91 | 1.26 | |
| Environmental concern high | | 1 if the respondent ranks above the median NEP | 0.72 | 0.45 | |

|  | **Definition of variables and summary statistics** *– Continued* | |  |  |  |
| --- | --- | --- | --- | --- | --- |
| Variables | Description | | Mean | Standard deviation |  |
| *Geographical proximity* | |  |  |  | |
| Berlin | | 1 if the respondent stems from Berlin | 0.15 | 0.36 | |
| *Socio-demographic characteristics* | |  |  |  | |
| Age | | The subject’s self−reported age. | 43 | 15 | |
| Female | | 1 if the subject reports being a woman. | 0.49 | 0.50 | |
| Highest education | | 1 if the subject’s highest level of education is at least secondary (Abitur in Germany). | 0.80 | 0.40 | |
| Crowdfunding in 2017 | | 1 if the subject reports that he/she contributed to a crowdfunding platform in 2017. | 0.51 | 0.50 | |

Appendix D: Robustness of estimation results

To investigate the robustness of the results, we added controls. Table D.1 presents discrete probability effects of treatments considering individual motivations and characteristics in the econometric analysis based on the parameters of the corresponding multinomial probit models. Table D.1 reveals robust results for the main treatment effects. Furthermore, individual green values were relevant for both the decision to donate and the allocation of donations. Environmental concern was positively correlated to overall donations, as the first column of the table documents. The higher the ranking of the NEP score, the higher the probability of donating money. This result is in line with previous findings showing an important role of environmental concern for pro−environmental behaviors (Kotchen and Moore, 2007; Attari *et al.,* 2009; Unsworth and Fielding, 2014; Fischbacher *et al.,* 2015; Ziegler, 2017). The next three columns further show that environmental concern was also related to the allocation of donations. An increase in environmental concern by one point was associated with a decreased estimated choice probability for donations to the crowdfunding platform by 3.32 percentage points and an increased choice probability of donating to the environmental organization by 5.18 percentage points. One explanation could be that the alternative environmental organization is more clearly associated with environmental activism, and thus, it could be perceived as more in line with the values and objectives of respondents with strong environmental concern. By contrast, green crowdfunding is highly heterogeneous in presenting its objectives, which is reflected in very different calls for action from project to project. Therefore, it is far less obvious that crowdfunding reflects the values and goals of a green individual. Participants feeling responsible for contributing to sustainable projects and startups, compared with respondents feeling not responsible, exhibited an increased choice probability for donating to the environmental organization by 5.92 percentage points. Our results reveal no statistically significant results on the relevance of further intrinsic individual motivations for overall donations and the allocation of donations.

The first column of Table D.1 further depicts that individuals exhibiting higher levels of social trust were more likely to donate the money than to make no contribution. This also corresponds to the effects of trust in charitable giving, as current research has shown (Taniguchi and Marshall, 2018; Neumayr and Handy, 2019). Moreover, social trust was correlated with the allocation decision, as the third column depicts. An increase in social trust by one point on the 11−point scale increased the choice probability for the environmental organization by 1.38 percentage points.

In line with findings from prior research that self−interested motivations reduce donations (Newman and Jeremy Shen, 2012; Chao, 2017), we found that material motives to support crowdfunding were statistically negatively correlated with overall donations. The first column in Table D.1 reveals that participants who strongly or very strongly agreed with the statement that they engage in crowdfunding because they seek a reward were less likely to donate than not donate. One explanation for this finding might be that the rewards offered on the crowdfunding platform were conditional on a donation, while the personal voucher was directly available. However, no statically significant results were found for the relevance of reward seeking for the allocative giving decision.

Regarding the social context factors, only the parameter for *Berlin community* exhibited a statistically significant effect on the allocation of donations. Respondents originating from Berlin, the location of the crowdfunding platform, exhibited an increased choice probability for donating to the crowdfunding platform by 11.47 percentage points and a decreased estimated choice probability for donations to the environmental organization by 10.34 percentage points. This geographical effect may be explained by a strong local community of green backers in Berlin, who are likely to be socially connected to the project−starters (Ordanini *et al.,* 2011; Castillo *et al.,* 2014; Agrawal *et al.,* 2015).

***Table D.1****: Average discrete probability effects by treatment group for contribution behavior − full specification*

|  | No contribution | Contribution to the crowdfunding platform | | Contribution to the environmental organization | | |  |  |
| --- | --- | --- | --- | --- | --- | --- | --- | --- |
| *Treatments (base: control group)* |  |  | |  | | |  |  |
| Writing task | −0.0289 | 0.1285** | | −0.0996** | | |  |  |
|  | (−0.95) | (2.51) | | (−2.12) | | |  |  |
| Recalling task | −0.0072 | 0.0152 | | −0.008 | | |  |  |
|  | (−0.22) | (0.29) | | (−0.16) | | |  |  |
| Picture | −0.0435 | 0.0613 | | −0.0179 | | |  |  |
|  | (−1.46) | (1.18) | | (−0.36) | | |  |  |
| Recalling task plus picture | −0.0211 | 0.1223** | | −0.1012** | | |  |  |
|  | (−0.65) | (2.36) | | (−2.14) | | |  |  |
| Writing task plus picture | −0.010 | −0.0091 | | 0.0191 | | |  |  |
|  | (−0.32) | (−0.17) | | (0.39) | | |  |  |
| *Motivations related to reputational concerns* |  | |  | |  | | |  |
| Expectation society | 0.0159 | | 0.0386 | | −0.0545 | | |  |
|  | (0.69) | | (0.95) | | (−1.43) | | |  |
| Behavior family and friends | 0.0028 | | 0.0059 | | −0.0087 | | |  |
|  | (0.14) | | (0.18) | | (−0.29) | | |  |
| *Benefits of giving* |  |  | |  | | |  |  |
| Reward seeking | 0.0510** | −0.0266 | | −0.0243 | | |  |  |
|  | (2.54) | (−0.75) | | (−0.75) | | |  |  |
| *Psychological benefits of giving* |  |  | |  | | |  |  |
| Warm glow feelings | −0.0144 | 0.0671 | | −0.0527 | | |  |  |
|  | (−0.59) | (1.53) | | (−1.31) | | |  |  |
| *Values and attitudes related to giving* |  |  | |  | | |  |  |
| Feeling responsible | −0.0297 | −0.0293 | | 0.0590* | | |  |  |
|  | (−1.45) | (−0.77) | | (1.69) | | |  |  |
| Local identification | −0.0152 | −0.0155 | | 0.0307 | | |  |  |
|  | (−0.79) | (−0.49) | | (1.06) | | |  |  |
| Social trust | −0.0125*** | | −0.0013 | | | 0.0139** | | |
|  | (−3.16) | | (−0.19) | | | (2.18) | | |
| *Green values* |  |  | |  | | |  |  |
| Environmental concern | −0.0183*** | −0.0344*** | | 0.0527*** | | |  |  |
|  | (−2.73) | (−2.64) | | (4.25) | | |  |  |
| *Geographical proximity* |  |  | |  | | |  |  |
| Berlin community | −0.0113 | 0.1128*** | | −0.1015** | | |  |  |
|  | (−0.43) | (2.64) | | (−2.57) | | |  |  |

| ***Table D.1*** *− Continued* | | | | | | | |
| --- | --- | --- | --- | --- | --- | --- | --- |
|  | No contribution | Contribution to the crowdfunding platform | | Contribution to the environmental organization | |  |  |
| *Socio-demographic characteristics* | | |  | |  | |  |
| Age | −0.0003 | | 0.0032*** | | −0.0029*** | |  |
|  | (−0.42) | | (3.00) | | (−3.00) | |  |
| Female | −0.0177 | | 0.0122 | | 0.0055 | |  |
|  | (−0.95) | | (0.39) | | (0.19) | |  |
| High education | −0.0399* | | 0.0169 | | 0.023 | |  |
|  | (−1.94) | | (0.44) | | (0.64) | |  |
| Crowdfunding in 2017 | −0.0011 | | 0.0084 | | −0.0073 | |  |
|  |  | |  | |  | |  |
| *Notes: The table reports the average discrete probability effects in the multinomial probit model using 1,008 observations. The dependent variable “Contribution behavior” includes the mutually exclusive alternatives: no contribution, contribution to the crowdfunding platform (base category), contribution to the environmental organization). Figures in parentheses are robust z−statistics. Coefficients that are statistically significant at 1% (5%, 10%) level are marked with *** (**, *), respectively.* | | | | | | |  |

Appendix E: Estimating Average Treatment Effects

Non−compliance to assigned treatment is prevalent in our experimental treatment groups 1, 2, 4, and 5. Participants could refuse to recall a happy event or to report a happy experience in the writing task. We filtered non−compliance using two strategies: first, we conducted content analyses of the answers for the treatments that contained the writing task (1 and 5). If they did not contain detailed information on the situation that made people happy, this was rated as non−compliance. We measured the time the respondents took to leave the treatment page for the treatments that only contained the autobiographical recall exercise (2 and 4). Following the typical times required in neuropsychological experiments for the performance of memory tasks that use episodic memory (Sestieri *et al.,* 2011), it was rated as non−compliance if respondents stayed less than 15 seconds**.** Since it was impossible to leave the treatment page before the picture was displayed, we did not observe any non−compliance in treatment group 3. Non−compliance is primarily an issue in the context of the recall task, as Fig E.1 shows. About half of the participants in treatments that contained the recall task complied.

***Figure D.1****: Treatment compliance by treatment (percent).*

We statistically addressed the potential bias due to non−compliance by opting for an instrumental variable (IV) regression approach (Angrist et al., 1996). Intention−to−treat (ITT) was used as an instrument for the proportion of compliants. This instrumental approach in the probit and multinomial probit model is estimated by the simulated maximum likelihood method (using 200 random draws in the underlying Geweke−Hajivassiliou−Keane (GHK) simulator). Table E.1 reports the simulated maximum likelihood estimations of the probit and multinomial probit model. It indicates the local average treatment effect (LATE) of affective primes on the allocative giving decision for those respondents who received the treatments. The pattern of the effect of emotions remains robust: Respondents who were invited to write down a happy event from the past year and those participants who were asked to recall a happy autobiographical event and were shown a picture simultaneously exhibited a significantly lower probability of donating to the environmental organization compared with the crowdfunding platform. In addition, considering the non−compliance issue, these participants were found to be significantly more likely to not donate and accept the voucher than to donate to the crowdfunding platform. Furthermore, in contrast to the results presented in Table 4, the recall task alone revealed a statistically significant effect on the allocation decision. The results above might thus be regarded as a lower bound of the estimated treatment effects.

**Table E.1***: Simulated maximum likelihood (ML) estimates of treatment assigned on contribution*

|  | Multinomial probit  Contribution behavior | |
| --- | --- | --- |
|  | No contribution | Contribution to the environmental organization |
| *Treatments assigned (base: control group)* |  |  |
| Writing task | −0.3510 | −0. 4692** |
|  | (−1.36) | (−2.30) |
| Recalling task | −0.4345 | −0. 5902** |
|  | (−1.29) | (−2.22) |
| Picture | −0.4370 | −0. 2675 |
|  | (−1.56) | (−1.28) |
| Recalling task plus picture | 0.7654** | −0. 5433** |
|  | (2.04) | (−2.09) |
| Writing task plus picture | 0. 0877 | 0. 0304 |
|  | (0.31) | (0.13) |
| Constant | −1.2302*** | −0.4331*** |
|  | (−8.09) | (−3.42) |
| *Notes: The table reports simulated maximum likelihood of parameters in the multinomial probit model using 1,008 observations. The dependent variable “Contribution behavior” includes the mutually exclusive alternatives: no contribution, contribution to the crowdfunding platform (base category), contribution to the environmental organization). Figures in parentheses are robust z−statistics. Coefficients that are statistically significant at 1% (5%, 10%) level are marked with *** (**, *), respectively.* | | |
