# Supplementary material for: Get the happiness out–An online experiment on the causal effects of positive emotions on giving
Source: PLoS One. 2023 Aug 23;18(8):e0290283. doi: 10.1371/journal.pone.0290283 (PMC10446232; doi:10.1371/journal.pone.0290283)
Supplement: Attachment — Submitted filename: Priming Response to Reviewer 2_final.docx [file pone.0290283.s002.docx]

**Response to Referee Report #2 from *PLOS ONE***

Get the happiness out – An online experiment on the causal effects of positive emotions on giving

*Manuscript number: PONE-D-22-32907*

Dear Reviewer,

Thank you very much for taking the time to give us guidance on improving our study “Get the happiness out – An online experiment on the causal effects of positive emotions on giving”. We value your thoughtful comments highly. We tried to incorporate them into the text and to respond to them as fully as possible. Please find our detailed responses to each suggestion (*in italics*) below. We worked through all of the comments and remarks, and we think that our paper has improved significantly as a result of us following them.

**Remark 1:** “Theory: The introduction discusses the theories of interest for this study, but without definition. To me, not being familiar with this area of psychology research, the intro was largely unintelligible to me, and I had to read the longer lit review more carefully to understand your framework.”

*We appreciate your comments regarding the introduction section's lack of definitions for the theories discussed in the paper. To address your point, we have introduced short definitions throughout the introduction and theory section to improve the overall readability but also intelligibility of our study for readers from other disciplines.*

**Remark 2:** “Theory: It seems to me that one of the big questions underpinning this line of inquiry is the causal direction - are happier people more likely to give, or does giving make you happy? I think you are trying to get at the former, by eliciting/reminding people of feelings of happiness before asking them to give. However, it still remains to be seen if those who did share a happy memory were more likely to give - can you qualitatively review the statements from the written task? But then, does having an easily recalled happy memory (does that actually make you happier?) make you more likely to give?”

*Thank you for your insightful comment. We have revised the introduction section to include part of your comment, which summarizes the reason for our inquiry more effectively than the previous version. We hope that this change has significantly improved the clarity and coherence of the introduction, and has addressed your concern.*

*Regarding the written statements: we evaluated them using a content analysis. We coded 1 if the individual shared information that could be interpreted as a description of a happy situation. Else it was coded as 0. This category included responses that made no linguistic sense, as well as responses that seemed to be too vague, completely unrelated, or stated, that they did not want to share private memories with us. Based on this evaluation we calculated the LATE effect.*

*Non−compliance to assigned treatment is prevalent in our experimental treatment groups 1, 2, 4, and 5. Participants could refuse to recall a happy event or to report a happy experience in the writing task. We filtered non−compliance using two strategies: first, we conducted content analyses of the answers for the treatments that contained the writing task (1 and 5). If they did not contain detailed information on the situation that made people happy, this was rated as non−compliance. We measured the time the respondents took to leave the treatment page for the treatments that only contained the autobiographical recall exercise (2 and 4). Following the typical times required in neuropsychological experiments for the performance of memory tasks that use episodic memory (Sestieri et al., 2011), it was rated as non−compliance if respondents stayed less than 15 seconds****.*** *Since it was impossible to leave the treatment page before the picture was displayed, we did not observe any non−compliance in treatment group 3. Non−compliance is primarily an issue in the context of the recall task, as Figure 1 shows. About half of the participants in treatments that contained the recall task complied.*

***Figure 1****: Treatment compliance by treatment (percent).*

Treatment compliance

|  | Treatment compliance - 15 seconds |
| --- | --- |
| Recalling task | 46.6667% |
| Recalling task plus image | 54.2169% |
| Writing task plus image | 92.3077% |
| Writing task | 93.9394% |
| Image | 100% |
| Control | 100% |

*We statistically addressed the potential bias due to non−compliance by opting for an instrumental variable (IV) regression approach (Angrist et al., 1996). Intention−to−treat (ITT) was used as an instrument for the proportion of compliants. This instrumental approach in* *the probit and multinomial probit model is estimated by the simulated maximum likelihood method (using 200 random draws in the underlying Geweke−Hajivassiliou−Keane (GHK) simulator). Table 2 of this review reports the simulated maximum likelihood estimations of the probit and multinomial probit model. It indicates the local average treatment effect (LATE) of affective primes on the allocative giving decision for those respondents who received the treatments. These results can also be found in Appendix E in the Online Appendix of the paper. The pattern of the effect of emotions remains robust: Respondents who were invited to write down a happy event from the past year and those participants who were asked to recall a happy autobiographical event and were shown a picture simultaneously exhibited a significantly lower probability of donating to the environmental organization compared with the crowdfunding platform. In addition, considering the non−compliance issue, these participants were found to be significantly more likely to not donate and accept the voucher than to donate to the crowdfunding platform. Furthermore, in contrast to the results presented in Table 4 in the paper, the recall task alone revealed a statistically significant effect on the allocation decision.*

**Table 2***: Simulated maximum likelihood (ML) estimates of treatment assigned on contribution*

|  | Multinomial probit  Contribution behavior | |
| --- | --- | --- |
|  | No contribution | Contribution to the environmental organization |
| *Treatments assigned (base: control group)* |  |  |
| Writing task | −0.3510 | −0. 4692** |
|  | (−1.36) | (−2.30) |
| Recalling task | −0.4345 | −0. 5902** |
|  | (−1.29) | (−2.22) |
| Picture | −0.4370 | −0. 2675 |
|  | (−1.56) | (−1.28) |
| Recalling task plus picture | 0.7654** | −0. 5433** |
|  | (2.04) | (−2.09) |
| Writing task plus picture | 0. 0877 | 0. 0304 |
|  | (0.31) | (0.13) |
| Constant | −1.2302*** | −0.4331*** |
|  | (−8.09) | (−3.42) |
| *Notes: The table reports simulated maximum likelihood of parameters in the multinomial probit model using 1,008 observations. The dependent variable “Contribution behavior” includes the mutually exclusive alternatives: no contribution, contribution to the crowdfunding platform (base category), contribution to the environmental organization). Figures in parentheses are robust z−statistics. Coefficients that are statistically significant at 1% (5%, 10%) level are marked with *** (**, *), respectively.* | | |

**Remark 3**: “Theory: I'm unsure about the option to give to the other environmental group, theoretically. What is that testing, or not testing?”

*The option to donate to the other environmental group was included in our study as an additional control condition, which allowed us to test the effectiveness of our intervention against a scenario where participants were given an alternative option to donate. In this control condition, participants were presented with the choice to donate to the other environmental group, which was not directly related to the cause we were promoting. This was intended to test whether our intervention was effective in stimulating donations to the target cause, or whether participants were simply more likely to donate in general.*

*We hope that this additional information clarifies the purpose of including the option to donate to the other environmental group. We emphasize the importance of addressing the question of whether incidental emotions trigger spillovers to other charities in the text accordingly on page 2.*

**Remark 4:** “Method: I couldn't really picture the setting/design of this experiment, and I read the appendix.”

*Thank you for your feedback. Figure 2 offers a comprehensive visualization of the experimental design, enhancing the clarity of the methodology. This flow diagram can also be found in Appendix A of the Online-Appendix accompanying the paper. If needed, we can add the figure directly to the paper.*

*Figure 2: Experimental design*

*Additionally, for improved clarity, we have included two screenshots that illustrate the survey deployment on the crowdfunding platform.*

*Screenshots: Survey implementation*

**4a)** “Are these people who were coming to the crowdfunding platform to make a contribution to one of the asks there (and thus already primed to give? - this may explain why they were much more likely to give over Bekkers, 2017).“

*Thank you for your question regarding the participants in our study. Table 1 provides a comparison of the proportion of donors and the mean amount contributed at the crowdfunding platform (not in the dictator game) by study participants and general visitors. It shows that around 10% of study participants donate to green crowdfunding projects, with contribution amounts ranging from €5 to €8,858 and an average contribution of €137.64 among those who give. If we exclude outlier values of €1,000 and €8,858, the average contribution of donors was €40.85 (Column 1 of Table 1).*

*Table 1: Proportion of donors and mean amount contributed among*

|  | *Study participants* | *Non-participants* | *Average visitor* |
| --- | --- | --- | --- |
|  | *(1)* | *(2)* | *(3)* |
| *Proportion of donors* | *9.8%* | *0.96%* | *3%* |
| *Average amount conditional on giving* | *€40.85* | *€72.52* | *€35* |

*Most donors in our sample contribute small amounts, which is typical for the crowdfunding context (Belleflamme et al., 2014). Approximately 75% contribute amounts up to €50 (see Figure 3), with only 18% donating between €50 and €100. Contribution amounts above €150 are extremely rare.*

*Figure 3:* *Contributions of donors in our sample and among visitors during data collection*

|  | Sample | Visitors during data collection |
| --- | --- | --- |
| up to 50€ | 75.25% | 62.55% |
| 50€ - 100€ | 17.82% | 22.88% |
| 100€ - 150€ | 3.96% | 4.8% |
| 150€- 200€ | 0.99% | 3.87% |
| 200€ - 1000€ | 0.99% | 5.17% |
| > 1000€ | 0.99% | 0.74% |

*When comparing our sample to non-participants, i.e. visitors to the crowdfunding platform during the data collection period who did not participate in the study (Column 2 of Table 1), we find that our sample has a higher proportion of donors. However, non-participants have larger donation amounts conditional on contributing than study participants, primarily due to the higher proportion of donations over €150, as shown in Figure 2. In addition, comparing the share of donors between study participants and average visitors to the crowdfunding platform (i.e. the average visitor from the perspective of the platform operators), reveals a similar picture (see Column 3 of Table 1). While study participants have a higher probability of contributing than average visitors, the donation amount conditional on contributing do not differ greatly between the two groups.*

*Overall, our sample may be more engaged with the platform compared to non-participants and average visitors, but our participants do not differ significantly in the typical amounts they donate. This is not surprising given that the totals include individuals that visit the site by accident, web crawlers, and other bots. Therefore, we can conclude that our data indicates that for practical purposes our participants most likely are human visitor that have a genuine interest in the projects hosted by the site and that are not more affluent or much more motivated to donate than other human visitors.*

*These results can be added to the paper, if needed.*

**4b)** “Were they then asked to complete this survey before they were able to access the crowdfunding projects? Can you clarify this?”

*Thank you for your question. Hopefully, we clarified the process of how visitors were offered the survey in the flowchart of Figure 2. In essence, all visitors to the crowdfunding platform were presented with the opportunity to participate in the online survey through a slider on the main page when they landed on the site from an external referrer URL If they rejected the survey, it was not offered again. If they did not take the survey immediately but did not reject it, it was offered again when they revisited the page. It is worth noting that completing the survey was not a prerequisite for accessing the crowdfunding projects on the site. Timestamps in our data revealed that those who participated, for the most, did so first before undertaking any further activities on the website, such as browsing projects or donating. The experimental design ensured that participants had the option to voluntarily decide whether to participate in the survey.*

**Remark 5**: “Method: I'm not sure what this as a "field experiment" over a standard "survey (lab style) experiment". There are pros and cons with each. Perhaps clarify that?”

*Thank you for your comment regarding the nature of our experiment. We understand your point because our experiment can be seen more as a "lab in the field" experiment rather than a field experiment in the classical sense. Our experiment shares some similarities with classical field experiments, such as selecting participants in their “natural” environment from the group of actual visitors of the crowdfunding platform and without their knowledge of being part of an experiment. The main difference between our experiment and classical field experiments is that the outcome is an "artificial" donation, as participants are asked to donate a sum they received as a quid pro quo measure for taking the survey. This feature reduces the external validity of our experiment, making it more similar to a classical survey experiment. We have added a reference to Gneezy and Imas (2017) to support this point and clarify the nature of our experiment. Additionally, we have replaced the term "field experiment" with "lab-in-field experiment" throughout the document to better reflect the experimental design.*

**Remark 6:** “Method: I have gotten used to seeing a graphic showing the treatment assignments in these types of papers (i.e. what happens when people enter the experiment).”

*We added the visual depiction of the experimental design, including treatment assignment, to the Online-Appendix A of the paper (see above).*

**Remark 7:** “Method: Did you give people the opportunity to opt out of the survey and continue to the crowdfunding platform? If so, can you describe the potential impact of selection bias in this situation?”

*Thank you for your question. We would like to clarify that participants in our study were given the option to decline the invitation to participate in the survey and continue to the crowdfunding platform. This ensured that participation in the survey was voluntary and not forced upon anyone. However, it is worth noting that participants who chose to opt out of the survey did not take part in the dictator game, which is the outcome variable in our study. Opting out may have introduced selection bias, and while we employed a randomized experimental design to mitigate its potential impact, this might still impact the results for instance by exacerbating ceiling effects. To address this limitation, we have added a discussion in the concluding remarks section of our paper as follows: “However, our study has several limitations. It reveals that online lab-in-field experiments suffer from strong homogeneity of the target audience of online platforms. The self-selection of participating in the experiment amplifies this limitation, as it can introduce correlations between causally unrelated background variables that co-determine a participant's susceptibility to positive emotions and willingness to donate in the dictator game. This effect could exacerbate ceiling effects that lead to null results, as observed in the present case.”*

*Further, referring to the comparison of the proportion of donors and the mean amount contributed at the crowdfunding platform (not in the dictator game) by study participants and general visitors shows that our participants differed in the extensive margin of their donation to the platform but not the intensive margin. The former has to be expected, as among all visitors among are, other things, bots and individuals who may have landed on the site by accident. All in all, the observed differences were minor and suggest that our participants are an acceptable representation of the average visitor who visits the platform out of genuine interest. Furthermore, the randomization process that we employed guards against collider effects that can arise due to self-selection into the study.*

**Remark 8:** “Method: Were they informed of the 5 Euro prior to starting the experiment? I'm not sure how that 'mitigates' the "experimenter demand effects". (p. 4).”

*Thank you for your feedback regarding our study's participation fee.*

*We feel that this comment is based on a misunderstanding. To clarify, participants were informed about the 5 Euro compensation at the beginning of the survey as a quid pro quo measure for their time and effort in completing the survey.* *Our purpose of mentioning the compensation was not to mitigate demander effects but to provide transparency regarding the study's compensation policy. Additionally, our study aimed to examine the impact of social distance on participants' donation behavior in the dictator game, which can inform our understanding of experimenter demander effects in future research.*

**Remark 9:** “Implications: The findings for this study seem important for understanding the various psychological mechanisms to incentivize volunteering, but I'd like to see some discussion about what the practical implications might be for practitioners. Asking donors to engage in some activity prior to giving may help spur these attitudes, but could also lead many, many donors to stop the process of completing a donation. CAn you”

*Thank you for your input. We greatly appreciate your suggestion, and we have taken it into consideration by including a new section on* *practical implications in the paper.*

*“**The finding that incidental positive emotions potentially affect donation behavior suggests that organizations seeking to increase charitable giving could benefit from incorporating activities or prompts that evoke positive emotions into their community-outreach processes. Charities can create opportunities for potential donors to get involved with the charity and feel a sense of closeness. This could include organizing volunteer events or social activities that bring people together and help them feel connected to the organization. For example, charities could create events that are enjoyable and fun, such as charity runs or auctions, to evoke positive emotions and create a sense of community among potential donors. Additionally, charities can offer social support to potential donors by providing resources or information that help them feel more connected to their cause. For example, offering groups or online forums for people interested in a particular issue can create a sense of familiarity and connection. Furthermore, practitioners could consider using digital media to engage with potential donors and create opportunities for them to share positive emotional experiences. Such strategies could help potential donors feel more connected to the charity and more likely to support the cause there than elsewhere.”*
