# Supplementary material for: Get the happiness out–An online experiment on the causal effects of positive emotions on giving
Source: PLoS One. 2023 Aug 23;18(8):e0290283. doi: 10.1371/journal.pone.0290283 (PMC10446232; doi:10.1371/journal.pone.0290283)
Supplement: Attachment — Submitted filename: Rebuttal letter to Reviewer 2.docx [file pone.0290283.s003.docx]

**Response to Referee Report #2 from *PLOS ONE***

Get the happiness out – An online experiment on the causal effects of positive emotions on giving

*Manuscript number: PONE-D-22-32907*

*Decision: Revision required [PONE-D-22-32907R1]*

Dear Referee,

Thank you again very much for taking the time to give us guidance on improving our paper “Get the happiness out – An online experiment on the causal effects of positive emotions on giving”. We value your thoughtful comments highly.

**Remark 1**: “However, the order of sections seemed odd - limitations the last paragraph, hypotheses after research design. I would structure the paper 1) Intro 2) Literature Review 3) Hypotheses 4) Research Design 5) Findings - Descriptive Statistics, then Analysis 6) Discussion 7) Conclusion”

*In response to your suggestions, we have made the necessary adjustments to address your concerns regarding the structure of the paper. Furthermore, we have conscientiously revised the discussion and conclusion sections, ensuring meticulous alignment with the prescribed criteria set forth by PlosOne.*

**Remark 2:** “Don't forget to really focus on the contribution your paper makes to the literature.”

*Wwe have explicitly addressed our contributions within the Discussion section.*
